# Early and delayed periprosthetic joint infection in robot-assisted total knee arthroplasty: a multicenter study

**DOI:** 10.1007/s00590-024-04043-0

**Published:** 2024-07-18

**Authors:** Carmelo Burgio, Francesco Bosco, Giuseppe Rovere, Fortunato Giustra, Giorgia Lo Bue, Antonio Petillo, Ludovico Lucenti, Gaetano Palumbo, Lawrence Camarda

**Affiliations:** 1 Department of Precision Medicine in Medical, Surgical and Critical Care (Me.Pre.C.C.), University of Palermo, Palermo, Italy; 2 Department of Orthopaedics and Traumatology, G.F. Ingrassia Hospital Unit, ASP 6, Palermo, Italy; 3 Department of Orthopaedics and Traumatology, Fondazione Policlinico Universitario A. Gemelli IRCCS, Università Cattolica del Sacro Cuore, Rome, Italy; 4 Department of Clinical Science and Translational Medicine, Section of Orthopaedics and Traumatology, University of Rome “Tor Vergata”, Rome, Italy; 5 Department of Orthopaedics and Traumatology, Ospedale San Giovanni Bosco di Torino-ASL Città di Torino, Turin, Italy; 6 Department of Orthopedic and Traumatology, Casa Di Cura Musumeci-GECAS, Catania, Italy

**Keywords:** Robot, RA-TKA, Total knee arthroplasty, PJI, Periprosthetic joint infection

## Abstract

**Background:**

Robot-assisted total knee arthroplasty (RA-TKA) has significantly improved knee surgery outcomes in the last few years. However, its association with the periprosthetic joint infection (PJI) rate remains debatable. This study investigates the incidence of early and delayed PJI in a multicentric cohort of patients who underwent RA-TKA, aiming to elucidate the risk associated with this procedure.

**Methods:**

This retrospective study analyzed data from a consecutive series of patients who underwent RA-TKA using the NAVIO Surgical System (Smith & Nephew, Memphis, USA) between 2020 and 2023. The inclusion criteria encompassed individuals over 18 years of age with a minimum follow-up period of three months. The primary outcome was the incidence of early and delayed PJI, defined according to the European Bone and Joint Infection Society (EBJIS) diagnostic criteria. Secondary outcomes included the evaluation of postoperative complications.

**Results:**

The study included patients who underwent RA-TKA with the NAVIO system, achieving an average follow-up of 9.1 ± 3.9 months. None of the patients met the EBJIS criteria for a likely or confirmed infection, indicating an absence of both early and delayed PJI cases. Two patients required subsequent surgical interventions due to patellar maltracking and prosthetic loosening, respectively. Additionally, three patients underwent passive manipulation under anesthesia (MUA).

**Conclusion:**

The findings indicate no evidence of early or delayed PJI in patients undergoing RA-TKA within the study period. The low complication rate further supports the reliability and safety of this surgical technique in short-term follow-up.

**Level of evidence:**

IV.

## Introduction

Total knee arthroplasty (TKA) has evolved significantly in recent years, becoming a prevalent procedure in orthopedic surgery [1–3]. Despite reports of substantial clinical success, dissatisfaction persists in approximately 20% of patients undergoing conventional TKA (C-TKA) [4, 5]. This has led to the development of Robot-Assisted (RA) TKA, a technique growing in popularity due to its precision in implant placement [6, 7]. RA-TKA utilizes preoperative X-ray or CT-scan images to tailor planning and implant selection to individual patient anatomy, potentially enhancing joint function, balance, and longevity [6]. The technique may also allow for more precise, minimally invasive approaches, resulting in less tissue damage and quicker recovery [7]. Early follow-up studies hint at improved outcomes [6], although RA-TKA is associated with longer surgical times, potentially increasing infection risk [6, 8–12].

Periprosthetic joint infection (PJI) remains a severe complication in TKA [8], leading to revisions, extended hospital stays, and increased costs, despite advancements in aseptic techniques and antibiotic management [13, 14]. Traditional TKA reports PJI incidences between 0.5% and 2.5%, making it a leading complication [13, 14]. In the U.S., infection is the primary cause of TKA revision failure, with an incidence of 44.1% [14]. However, the incidence of PJI in RA-TKA is not well-documented in the literature [12–14].

Diagnosing PJI is complex, with multiple criteria developed over time [15, 16]. The ICM 2013 criteria have been historically prevalent [17], and in 2018, more sensitive criteria were introduced, though not universally accepted [18]. Both criteria have limitations in confirming or excluding infection [17, 18]. In response, the European Bone and Joint Infection Society (EBJIS) introduced new PJI criteria in 2021 [19], featuring a middle category for patients with a higher likelihood of infection and proposing a staged diagnostic approach, using less invasive tests initially, followed by more specific tests if needed. This approach has shown promise in improving diagnostic accuracy [19–22].

The primary aim of this study was to assess the incidence of early PJI following RA-TKA using the EBJIS criteria. Additionally, the study evaluated the occurrence of delayed PJI in a select group of patients. A comprehensive analysis of postoperative complications was also conducted to provide a thorough evaluation of this patient population. The hypothesis posits that RA-TKA is not associated with an elevated risk of early or delayed PJI.

## Material and methods

### Study design

This retrospective study examines a consecutive series of patients diagnosed with grade III-IV knee osteoarthritis, classified according to the Ahlbäck classification, who underwent RA-TKA at the Orthopedic and Traumatology Unit of AOUP 'P. Giaccone', Palermo, Italy, and at Casa Di Cura Musumeci-GECAS, Catania, Italy, between January 1, 2020, and September 1, 2023. Surgical procedures were conducted by two experienced surgeons (L.C. and G.P.), and all patients received a cemented prosthesis. Antibiotic prophylaxis followed standard protocols, with cefazolin administered unless contraindicated by beta-lactam allergy, MRSA history, or current MRSA colonization, in which case vancomycin or a broad-spectrum antibiotic was used [23].

All patients received standard postoperative care at their respective institutions, including early mobilization, active and passive range of motion (ROM), and weight-bearing as tolerated, immediately postoperatively. Patients were discharged after an average of 5 days, ranging from four to seven days. Discharge criteria included effective pain control, knee flexion of at least 90°, and independent mobilization with crutches.

The follow-up protocol consisted of a thorough evaluation during the first year after surgery, involving both clinical and radiological assessments. Clinical evaluations were conducted on the fifteenth day, first month, second month, third month, sixth month, and first year post-surgery. Postoperative X-rays were obtained at one month, three months, and one-year postoperatively.

### Clinical evaluation

Two researchers (C.B. and F.B.) conducted the follow-up clinical assessments. This evaluation included a thorough review of each patient's recent pharmacological and medical history, focusing on wound healing issues, fever, purulence around the prosthesis, and the presence of any sinus tract. The assessment also encompassed a detailed observation of symptoms such as erythema at the surgical site, swelling, warmth, pain around the surgical wound, and a comprehensive recording of all postoperative complications.

### Radiographic analysis

Anteroposterior (AP), lateral, and AP long-leg weight-bearing radiographs of the knee were conducted to evaluate potential radiological signs of implant loosening. The radiographs were carefully evaluated by an expert orthopedic surgeon (L.L.) for the presence of any radiolucent lines at the bone-implant interface, osteolysis, or changes in implant position that could indicate loosening of the prosthetic components. In case of any uncertainties, a third author (L.C.) was involved to resolve any disagreements.

### Inclusion and exclusion criteria

Inclusion criteria for the study included utilizing the NAVIO Surgical System (Smith & Nephew, Memphis, USA) for knee replacement surgery, age greater than 18 years and a minimum follow-up period of at least three months. Exclusion criteria were history of ipsilateral UKA or revision TKA, contralateral TKA, knee osteotomy, ligamentous reconstruction, or extensive trauma-related surgery.

### PJI diagnostic criteria

The occurrence of PJI is typically classified into three postoperative phases: early, delayed, and late [17, 18]. The early phase is defined as the period within three months following surgery, the delayed phase extends from just over three months to within 12 months, and the late phase occurs more than 12 months after the surgical procedure [18]. Our study primarily focuses on the immediate postoperative period, a critical timeframe for evaluating postoperative risks and complications associated with RA-TKA. This accurate evaluation is vital for assessing the initial response to the surgical procedure, the effectiveness of infection control measures, and the overall immediate safety profile of the RA technique [18].

**Fig. 1:**
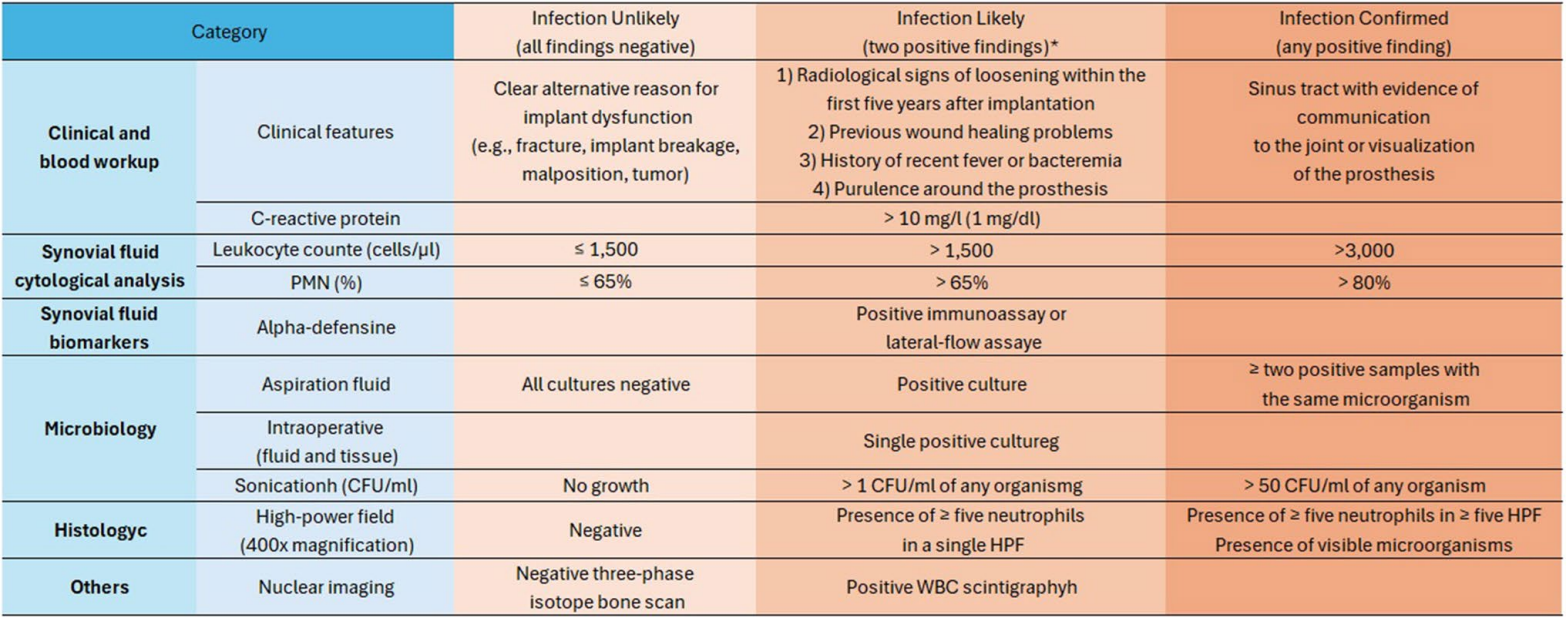
EBJIS criteria for the diagnosis of periprosthetic joint infection (PJI). *—Infection is only likely if there is a positive clinical feature or raised serum C-reactive protein (CRP), together with another positive test (synovial fluid, microbiology, histology, or nuclear imaging). PMN—Polymorphonuclear; %—percentage; CFU—Colony Forming Unit; WBC—White blood cells

The EBJIS has established diagnostic criteria to standardize the diagnosis of joint infections, specifically focusing on PJIs. Based on these guidelines, three diagnostic categories are identified: 'Infection Unlikely', 'Infection Likely', and 'Infection Confirmed' [19–21] (Fig. 1).

In this study, the suspicion of PJI was raised based on the EBJIS clinical criteria, specifically under the classifications of "Infection Likely" or "Infection Confirmed," within three months and one-year postoperatively, for early and delayed PJI, respectively [19, 20]. Patients who responded positively to the “Infection Likely” and “Infection Confirmed” clinical criteria were requested to return to the hospital for further evaluation. This evaluation encompassed the examination of serum C-reactive protein (CRP), synovial fluid analysis, microbiology, histology, and nuclear imaging. According to EBJIS diagnostic criteria [19], the research team ensured that these patients had no other inflammatory causes, immunosuppressive conditions, or antibiotics and immunosuppressant intake within two weeks before the examination that could affect the sensitivity and specificity of these inflammatory markers.

### Data collection

Patients were stratified according to their minimum follow-up: early, for patients with a minimum follow-up of three months, and delayed, for patients with a minimum follow-up of one-year. Data analysis was managed by an independent author (C.B.). In cases, where data discrepancies or uncertainties arose, an experienced knee surgeon (L.C.) was consulted to ensure resolution and accuracy. Any cases of disagreement were resolved by a third author (F.B.).

### Data extraction

This study enrolled 137 patients who underwent RA-TKA: 66 patients received treatment at the Orthopedic and Traumatology Unit, AOUP 'P. Giaccone', Palermo, Italy, and 71 at Casa Di Cura Musumeci-GECAS, Catania, Italy. Participants were selected based on a uniform assessment process, evaluating demographic information, medical history, and follow-up duration to align with predefined inclusion criteria. Out of the initial cohort, 19 patients were excluded because considered lost to follow-up. This categorization was based on their discontinuation of participation in scheduled outpatient check-ups and subsequent nonresponsiveness to multiple contact attempts via telephone and email. Consequently, 118 patients met all the inclusion criteria and were declared eligible for the early PJI evaluation.

**Fig. 2:**
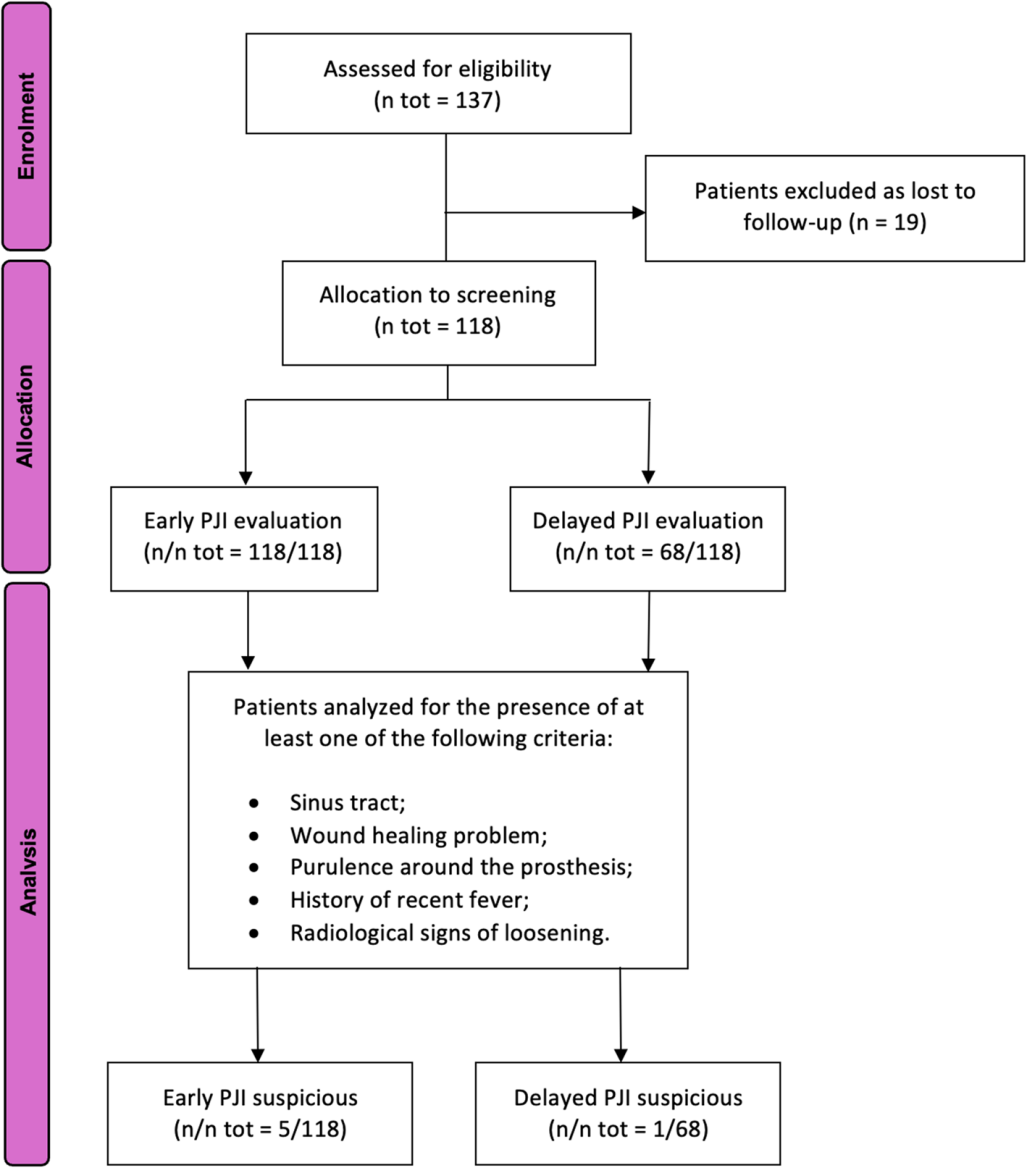
Flowchart of patient enrollment in the study: a comprehensive overview. n—number of evaluation cases; n tot—total number of evaluation cases; EBJIS—European bone and joint infection society; PJI—periprosthetic joint infection

At the final follow-up, 68 patients were found to have a minimum follow-up of one-year. Therefore, a delayed PJI investigation was carried out for these patients. The detailed patient enrolment process is shown in the flow chart (Fig. 2).

### Ethical approval

This retrospective study, examining an established surgical procedure, strictly adhered to the ethical guidelines set forth in the 1964 Helsinki Declaration and its subsequent amendments. All participating patients were fully informed about the nature and purpose of the study and provided their informed consent before than the surgical procedure. Research Protocol: Robotic Knee Prosthesis. Approval by Local Ethics Committee Palermo 1: Minutes No. 03/2024.

### Statistical analysis

The statistical analysis of the data involved expressing categorical variables as counts and percentages and continuous variables as means with standard deviations (SDs). The statistical analyses were performed using IBM SPSS Statistics version 26.0 (IBM Corp., Armonk, NY, USA), allowing for a comprehensive and accurate interpretation of the study's findings.

## Results

### Demographic and clinical data

After applying inclusion and exclusion criteria, a total of 118 patients were recruited and analyzed. The cohort comprised 74 women (62.7%) and 44 men (37.3%). The median follow-up period was 9.1 ± 3.9 months. The average age at follow-up was 68.9 ± 8.1 years, and the average body mass index (BMI) was 29.7 ± 5.6 kg/m2. The average surgical time was 121 ± 20.5 min. Prostheses were more commonly placed on the right side in 61 patients (51.7%), while 57 patients (48.3%) had left-sided prostheses. Detailed demographics are shown in Table 1.

**Table 1:** Demographic and clinical data of patients enrolled in the study cohort

Demographic characteristics	Values
Patients, Total N°	118
*Gender*	
Male, N° (%)	44 (37.3)
Female, N° (%)	74 (62.7)
Age, (years old), Mean ± SD	68.9 (8.1)
*BMI (kg/m2)*	
Male, Mean ± SD	29.3 (4.0)
Female, Mean ± SD	29.9 (6.4)
Total, Mean ± SD	29.7 (5.6)
Follow-up time (months), Mean ± SD	9.1 (3.9)
Surgical timing (minutes), Mean ± SD	121 (20.5)
*Side of prosthesis*	
Right, N° (%)	61 (51.7)
Left, N° (%)	57 (48.3)
*Comorbidities*	
Diabetes, N° (%)	20 (16.9)
Hypertension, N° (%)	16 (13.6)
Vascular disease, N° (%)	11 (9.3)
Peripheral neuropathy, N° (%)	7 (5.9)
Rheumatic disorders, N° (%)	9 (7.6)
Fracture, N° (%)	4 (3.9)

N°—number of evaluation cases; SD—standard deviation; %—percentage, Kg—kilogram; m—meter

### Early PJI evaluation

A significant observation was that none of the patients fulfilled the EBJIS criteria for “Infection Likely” or “Infection Confirmed” in the early PJI evaluation. This is noteworthy, especially considering that the average C-reactive protein (CRP) levels in the five suspected cases of infection were 1.5 ± 0.5 mg/dL, which falls within normal limits and does not suggest an infection-related inflammatory response. The synovial fluid cytological analysis revealed a leucocyte count of less than 1,500 cells/µl and a percentage of polymorphonuclear neutrophils (PMN) below 65% in all five patients examined. The average surgical timing of patients suspected of early PJI was 119.4 ± 2.9 min, slightly lower than the population’s average. The most common EBJIS clinical sign for the suspicion of early PJI was wound healing problems, observed in five patients (4.2%). Specifically, three patients showed prolonged diastasis of the margin, and two patients presented bleeding in the upper and middle-lower part of the wound. All wound healing problems were adequately addressed with dressing, and no surgical wound revision was needed. Furthermore, radiological assessments revealed no indications of prosthetic loosening, a common sign of PJI.

### Delayed PJI evaluation

Radiological signs of loosening were detected at the one-year follow-up for one patient. Subsequently, suspicion of delayed PJI was raised, and the patient underwent further examinations to fulfill diagnostic criteria. Serum C-reactive protein levels were within the normal range, indicating an absence of an inflammatory response. Synovial fluid analysis appeared clear with no bacterial growth detected. Histological examination, as well as nuclear imaging, did not present any significant inflammatory response. As a result, the diagnosis of delayed PJI, according to EBJIS diagnostic criteria of “Infection Likely” or “Infection Confirmed”, was excluded. Furthermore, no particular differences were seen in the surgical timing compared to the study cohort.

As shown in Table 2, these findings emphasize the non-occurrence of delayed PJI among the patients who underwent RA-TKA within one-year of follow-up. This outcome highlights the potential effectiveness of RA-TKA in mitigating short-term postoperative joint infection risks.

**Table 2:** Postoperative characteristics of patients with PJI suspicion

Patients	Age (years)	Timing of clinical PJI suspicious	Surgical timing (minutes)	E	S	W	P	Recent fever	Wound healing problem	Purulence around the prosthesis	Sinus tract	Radiological sign of loosening	CRP (mg/dL)
1	79	30 days	117	Y	Y	Y	Y	N	Y	N	N	N	1,5
2	71	15 days	120	N	Y	Y	Y	N	Y	N	N	N	1,3
3	71	30 days	116	N	Y	N	Y	N	Y	N	N	N	0,8
4	52	90 days	121	N	Y	Y	Y	N	Y	N	N	N	2,2
5	70	60 days	123	Y	Y	Y	Y	N	Y	N	N	N	1,7
6	62	1 year	115	Y	Y	Y	Y	N	N	N	N	Y	1.1

E—Erythema of the surgical site; S—Swelling; W—Warmth; P—Pain in or around the surgical site; CRP—C-reactive protein; Y—yes; N—no; mg—milligram; dL—deciliter

### Postoperative complications and clinical outcome

The study found few significant complications, with only two cases necessitating additional surgical procedures. One patient experienced patellar maltracking and underwent revision five months after the primary procedure. The second patient underwent revision one-year postoperatively due to prosthetic loosening attributed to the malposition of the tibial component, following the exclusion of infectious causes based on EBJIS criteria. Additionally, three individuals underwent passive manipulation under anesthesia (MUA) to address knee stiffness and limited ROM, occurring at four, seven, and eight months postoperatively, respectively. Notably, there were no reported instances of wound infection.

## Discussion

The study's main finding was that RA-TKA performed using the NAVIO surgical system did not result in any cases of PJI, as defined by the EBJIS criteria [19]. Specifically, no cases were classified as “Infection Likely” or “Infection Confirmed” for early or delayed PJI. Two patients required secondary surgical procedures related to different aseptic complications, at five months and one-year postoperatively. This suggests that, while certain complications were noted, short-term postoperative joint infection risks do not seem to be increased by RA-TKA.

While, traditional TKA has been reported to yield generally good clinical results, dissatisfaction rates as high as 20% have been documented [4, 5]. RA-TKA was developed to address this issue, offering enhanced precision and accuracy in implant placement. Smith et al. [9] observed a higher patient satisfaction rate of 94% in RA-TKA compared to 82% in manual TKA. Gao et al. [10] conducted a meta-analysis revealing that kinematic alignment in RA-TKA could lead to better clinical outcomes than mechanical alignment in the short-term. Similarly, Zhang et al. [11] demonstrated improved accuracy in component positioning and better patient-reported outcomes in RA-TKA versus manual TKA.

Our study contributes to the limited assessment of PJI risk in RA-TKA. We observed no increased risk of PJI in RA-TKA, and this evidence aligns with other research comparing PJI rates between RA-TKA and C-TKA [24–28].

For instance, Vandenberk et al. [27] published clinical results of a single-center retrospective cohort study of 230 NAVIO RA-TKA patients and 489 C-TKA patients, with a mean follow-up of 31 months and 29.7 months, respectively, that resulted in a PJI rate of 0% for RA-TKA compared to 1% for C-TKA. Joo et al. [26] reported a PJI rate of 0.2% in 851 patients who underwent RA-TKA, with a follow-up ranging from 4 to 15 months. Mitchell et al. [28] performed a comparative study between RA-TKA and C-TKA, describing no statistically significant difference in complication rates at one-year follow-up.

The detailed planning and multiple steps involved in RA-TKA with robotic systems contribute to increased surgical time. Studies have consistently shown prolonged surgical durations in RA-TKA compared to C-TKA [12, 26–28]. In their research, Nogalo et al. [12] concluded that RA-TKA is associated with longer operative times. Given the extended surgical duration, there has been a growing focus on related complications, particularly the risk of developing PJI. Pugely et al. [29] identified an increased risk of infection after 120 min of surgery, while Peersman et al. [30] and Ravi et al. [31] also reported increased infection risks associated with longer TKA surgeries. However, these findings are not universally consistent. Our study's average surgical time was 121 ± 20.5 min, similar to other studies [32–34], which did not report an increased PJI rate despite extended surgical durations. Specifically, Singh et al. [32], Held et al. [33], and Scigliano et al. [34] found no significant difference in postoperative complications or revision surgery rates due to prolonged surgical times.

The main strengths of this study include its considerable cohort size, which enhances the level of evidence. The multicentric approach broadens patient diversity, ensuring more robust and generalizable data. Additionally, the meticulous application of exclusion criteria, particularly for patients with alternate sources of inflammation or immunodepression, significantly bolsters the validity of CRP measurements in the context of assessing surgical outcomes.

However, this study is not without limitations. Firstly, its retrospective design inherently carries biases typical of such methodologies, which might affect the interpretation of the results. Secondly, despite the implementation of uniform peri-operative protocols across different centers, subtle variations in surgical techniques could potentially impact clinical outcomes. The third limitation of the study lies in the absence of a control group, which is crucial for a comprehensive evaluation of the impact of independent variables. A control group enables the isolation of specific effects, necessitating caution in interpreting the obtained results. Lastly, the follow-up duration constraints limited our ability to assess late PJIs. The study also did not fully encompass the evaluation of delayed PJIs due to the inability to achieve a complete one-year follow-up for all participants. To mitigate this limitation, a rigorous and ongoing follow-up protocol has been instituted for the included patients. These continued assessments are essential for the prospective monitoring and detection of delayed and late PJIs, providing a more comprehensive understanding of the long-term infection risks associated with the surgical procedure.

## Conclusion

This study demonstrates the absence of early and delayed PJI in RA-TKA cases, using the EBJIS criteria, within the study period, thereby contributing additional evidence to this technique’s short-term efficacy and safety. However, further research is necessary to corroborate these findings and to confirm the long-term reliability of RA-TKA, particularly in the context of late PJI.

## Data Availability

Dataset analyzed in this study is available from the corresponding author on reasonable request.
